# Pro-environmental behaviors: the role of critical thinking and emotional awareness in shaping sustainable practices

**DOI:** 10.3389/fpsyg.2026.1730693

**Published:** 2026-04-10

**Authors:** Rosa Angela Fabio, Laura Manoti

**Affiliations:** Department of Biomedical and Dental Sciences and Morpho-Functional Imaging, University of Messina, Messina, Italy

**Keywords:** critical thinking, emotional avoidance, emotional awareness, pro-environmental behavior, sustainability

## Abstract

**Introduction:**

Critical thinking is increasingly recognized as a key competence for addressing global challenges, including sustainability. At the same time, emotional avoidance–particularly reduced awareness of negative emotions related to climate change–may undermine engagement in environmentally relevant actions.

**Methods:**

The present study examined the joint role of critical thinking and emotional awareness in shaping self-reported pro-environmental behaviors, using sustainable practices as the primary outcome. A sample of 309 adults (59.5% female; Mage = 31.5, SD = 11.7) completed validated measures of critical thinking dispositions, emotional avoidance, and sustainable behaviors.

**Results:**

Correlational analyses indicated that critical thinking was positively associated with sustainable behaviors (*r* = 0.34, *p* < 0.001), whereas lack of emotional awareness was negatively associated (*r* = −0.27, *p* < 0.01). Regression analyses further showed that higher critical thinking independently predicted greater engagement in sustainable behaviors, while reduced emotional awareness predicted lower engagement.

**Discussion:**

These findings suggest that fostering critical thinking and emotional awareness may support engagement in pro-environmental behavior. The study adopts a Theory of Planned Behavior-informed perspective to frame the investigation of psychological factors associated with self-reported sustainable behaviors, while acknowledging that no TPB model was directly tested.

## Introduction

Critical thinking is widely recognized as a core competence for solving problems, making decisions, and evaluating evidence in a reasoned and systematic way (Willingham, 2007; Sternberg, 2018; Paul and Elder, 2020). It enables individuals to consider multiple perspectives, remain open to disconfirming information, and draw conclusions that are logically consistent with the available data (Profetto-McGrath et al., 2009). While conceptualizations vary, scholars consistently emphasize the processes of clarification, analysis, evaluation, inference, and self-monitoring as central components of critical thinking (Facione, 2015; Fabio et al., 2025a).

Beyond these processes, critical thinking also involves a deep understanding of information and the appropriate application of reasoning skills (Halpern and Dunn, 2022). Importantly, these abilities are not confined to academic contexts but are increasingly relevant in professional, social, and ecological domains (Verburgh et al., 2013; Ahuja et al., 2023; Javaid et al., 2023).

In this study, critical thinking is operationalized using the Critical Thinking Attitude Scale (CTAS; Facione et al., 1995; Fabio et al., 2025a), which measures four dimensions: Systematicity, Analyticity, Truth-Seeking/Open-Mindedness, and Intellectual Curiosity. This allows us to link theoretical frameworks of critical thinking directly to measurable constructs in our study.

At the same time, the environmental crisis represents one of the most urgent global challenges, as human activity drives climate change, biodiversity loss, and large-scale ecological degradation (Ripple et al., 2017). Addressing these issues requires profound changes in behavior (Nielsen et al., 2020), making it critical to understand the psychological factors that drive engagement in environmentally responsible actions.

Research in environmental psychology has examined a wide range of pro-environmental behaviors (PEBs)—such as recycling, water and energy conservation, and participation in ecological initiatives—that directly contribute to the protection of natural resources (Howard, 2000; Otto et al., 2014; Vesely et al., 2022). Closely related, but conceptually distinct, are sustainable consumption behaviors (SCBs), which focus on reducing the ecological impact of products and services across the stages of purchase, use, and disposal, balancing environmental, social, and economic consequences (White et al., 2019; Luchs et al., 2010; Wang and Udall, 2023).

The Theory of Planned Behavior (TPB; Ajzen, 1991, 2005, 2012) remains one of the most influential models for explaining such behaviors. According to the TPB, attitudes, subjective norms, and perceived behavioral control jointly shape intentions, which in turn predict action. Empirical evidence supports the predictive validity of these determinants across various pro-environmental contexts (Bosnjak et al., 2020; Tilikidou and Delistavrou, 2014). Yet, despite its robustness, the TPB leaves open important questions regarding the cognitive and emotional processes that may strengthen—or weaken—the translation of ecological awareness into sustainable behavior. In particular, the TPB provides limited specification of the cognitive and emotional processes that precede and inform attitude formation.

In the present study, critical thinking and emotional awareness are conceptualized as cognitive-emotional resources that are theoretically informed by the TPB framework, rather than as components of an empirically tested TPB model. Rather than functioning as direct antecedents of behavior or as boundary conditions of intention formation, these resources are proposed to influence how environmental information is evaluated and emotionally processed, thereby shaping engagement in self-reported pro-environmental behaviors. Accordingly, the present study adopts a TPB-informed perspective, without testing or extending the TPB model itself.

With reference to critical thinking, scholars argue that sustainable decision-making requires the ability to analyze complex environmental issues, evaluate diverse perspectives, and reason beyond immediate self-interest (Lamb et al., 2017; Lange and Dewitte, 2019). Critical thinking supports such competencies, fostering openness to evidence, awareness of long-term consequences, and autonomy in decision-making (Rhodes, 2010; Nawawi et al., 2024). Recent contributions also highlight its growing role in sustainability education, where it is considered a key resource for promoting ecological awareness and responsible behavior (Felix and Clayton, 2025). Nevertheless, empirical evidence directly linking critical thinking to PEBs remains scarce, leaving a theoretical gap in understanding its specific contribution.

With reference to the regulation of negative emotions, several studies show that individuals often avoid or suppress climate-related emotions such as anxiety, guilt, or fear, a strategy that reduces cognitive elaboration and undermines motivation for sustainable action (Lertzman, 2015; Clayton, 2020). Avoidant coping mechanisms—such as denial or minimization of climate threats—are associated with disengagement and weaker pro-environmental intentions (Norgaard, 2011; Stoknes, 2015). In contrast, when processed constructively, emotions like concern can facilitate engagement and sustained behavioral commitment (Ojala, 2012; Stanley et al., 2021). Thus, emotional avoidance may act as a psychological barrier, weakening the link between awareness of environmental risks and actual ecological behaviors (Reser and Bradley, 2020).

Against this background, despite the extensive literature on pro-environmental behavior and the widespread use of the Theory of Planned Behavior, less attention has been devoted to the cognitive and emotional processes that operate prior to attitude formation. In particular, empirical research has rarely examined critical thinking as a measurable psychological resource directly associated with sustainable behavior, and existing studies tend to overlook how individuals cognitively evaluate and emotionally process environmental information in relation to their engagement in sustainable behaviors. Moreover, although pro-environmental behavior represents a broad behavioral domain, sustainable consumption behaviors constitute a more specific and observable subset that captures everyday ecological engagement. In the present study, critical thinking is operationalized through the Critical Thinking Attitude Scale as a dispositional cognitive resource, while sustainable behaviors are examined as the primary behavioral outcome. By integrating critical thinking and emotional awareness within a TPB-informed perspective, this study addresses a theoretical and empirical gap by clarifying how cognitive–emotional resources are associated with engagement in sustainable behavior.

## The present study

Based on a TPB-informed perspective, the present study examines whether critical thinking and emotional awareness are associated with self-reported sustainable behaviors. Rather than testing indirect effects through attitudes or intentions, the study focuses on behavioral outcomes, acknowledging that TPB core constructs were not directly measured.

H1: Higher levels of critical thinking will positively predict sustainable behaviors, indicating that greater analytic and evaluative thinking is associated with stronger engagement in pro-environmental actions (Facione, 2015; Fabio et al., 2024; Fabio and Croce, 2024; Vesely et al., 2022).

H2: Greater lack of emotional awareness will negatively predict sustainable behaviors, such that reduced awareness of negative emotions is associated with lower engagement in pro-environmental actions (Lertzman, 2015; Clayton, 2020; Norgaard, 2011; Stoknes, 2015; Reser and Bradley, 2020; Stanley et al., 2021).

This study therefore contributes to the literature by examining cognitive and emotional correlates of sustainable behavior within a TPB-informed framework. Specifically, critical thinking is conceptualized as a cognitive resource associated with how individuals evaluate and integrate environmental information, while emotional awareness supports the processing of climate-related emotions. Together, these cognitive–emotional resources are expected to be associated with everyday engagement in sustainable practices, without implying a direct test or extension of the Theory of Planned Behavior.

## Method

### Participants

An a priori power analysis was conducted using GPower 3.1 (Faul et al., 2009) to estimate the minimum sample size required to detect medium-sized effects in the relationships among the study variables. Because the study's primary models involved a small number of predictors, the power analysis was based on detecting a medium-sized bivariate association (*r* = 0.30) as a conservative benchmark, rather than on a full hierarchical regression specification. Following conventional recommendations in psychological research, a two-tailed significance level of α = 0.05 and desired statistical power of 1–β = 0.80 were assumed. Under these parameters, the analysis indicated a minimum required sample size of *n* = 84.

Although the main hypotheses were tested using multiple linear regression analyses, the final sample size (*N* = 309) substantially exceeded the minimum requirement, ensuring adequate statistical power for the regression models reported and allowing for the detection of smaller effects and more stable parameter estimates. Participants were eligible if they were 18 years of age or older, resident in Italy, and able to complete the questionnaire in Italian. No additional exclusion criteria were applied. Data were collected between March and June 2024.

### Sampling method and recruitment

Participants were recruited using a convenience sampling strategy. The survey link was disseminated via email and online contacts, including university mailing lists and the personal and professional networks of the research team. Participation was voluntary and anonymous, and no incentives were provided. To reduce the likelihood of duplicate responses, the online survey platform restricted participation to one submission per device and browser session. Only fully completed questionnaires were retained for analysis. This sampling approach was considered appropriate given the exploratory nature of the study and its focus on examining psychological relationships rather than estimating population parameters. Because the survey was anonymous and distributed online, an exact response rate could not be calculated; however, incomplete questionnaires were excluded prior to analysis.

## Measures

### Brief Experiential Avoidance Questionnaire (BEAQ)

The Brief Experiential Avoidance Questionnaire (BEAQ; Gámez et al., 2014) is a 15-item self-report measure assessing the tendency to avoid or escape unwanted internal experiences, such as emotions, thoughts, and bodily sensations. Participants responded on a 6-point Likert scale (1 = strongly disagree to 6 = strongly agree). In the present study, three scores were computed: Tendency to Avoid Emotions, Lack of Emotional Awareness During Activity, and a Total Experiential Avoidance score. These scores reflect the operationalization adopted in the present analyses. Higher scores indicate greater experiential avoidance. In the current sample, the BEAQ showed adequate internal consistency (Cronbach's α = 0.78–0.89).

### Critical Thinking Attitude Scale (CTAS)

The Italian version of the Critical Thinking Attitude Scale (CTAS; Facione et al., 1995; Fabio et al., 2025a) was used to measure participants' disposition toward reflective, analytical, evaluative, and metacognitive thinking. The CTAS consists of 26 items across four subscales: Systematicity (9 items), Truth-Seeking and Open-Mindedness (6 items), Analyticity (4 items), and Intellectual Curiosity (7 items). Participants responded on a 7-point Likert scale ranging from 1 (strongly disagree) to 7 (strongly agree), with higher scores indicating stronger critical thinking attitudes. Example items include “I can think in an analytic way” (Analyticity), “I remain focused when solving a problem” (Systematicity), “I always seek the most reliable sources to understand a problem” (Truth-Seeking and Open-Mindedness), and “I have a strong desire to learn new things” (Intellectual Curiosity). The CTAS has shown good internal consistency, with Cronbach's alpha values above 0.78 across factors.

### Sustainable Behaviors Scale (SBS)

The Sustainable Behaviors Scale (SBS; Luchs et al., 2010; Fabio et al., 2025b) was used to measure sustainable practices. The SBS comprises 18 items rated on a 7-point Likert scale (1 = Never to 7 = Always). It includes four subscales: Sustainable Purchasing and Awareness (8 items), Reuse and Recycling (4 items), Reduction (4 items), and Walking and Public Transportation (2 items). Example items are “I regularly use public transportation, bicycle, or walk instead of driving alone” (Walking and Public Transportation), “Whenever possible, I buy second-hand clothing” (Sustainable Purchasing and Awareness), “I recycle plastic using appropriate containers” (Reuse and Recycling), and “When at home, I turn off lights when leaving a room” (Reduction). The SBS showed good internal consistency for the total scale (α = 0.85).

All measures were selected based on their established psychometric properties and their conceptual relevance to the study objectives.

### Procedure

The invitation briefly described the study aims and included a link to the online questionnaire hosted on Google Forms. The survey link was disseminated via email and online contacts, including university mailing lists and the personal and professional networks of the research team.

Before beginning the questionnaire, all participants were presented with an information sheet describing the study's aims, procedures, and their rights, including confidentiality and the option to withdraw at any time without penalty. Informed consent was obtained electronically prior to participation. On average, completion of the survey required approximately 20 min.

The study protocol was reviewed and approved by the Ethics Committee of the University of Messina [CERIP; Prot. n. 0019672, 09/03/2025–(UOR: SI001165–Classif. III/14)].

### Statistical analysis

All analyses were conducted using IBM SPSS Statistics 25. Descriptive statistics (means, standard deviations, observed ranges, skewness, and kurtosis) and reliability coefficients (Cronbach's α) were computed for all measures. Pearson's correlation coefficients were calculated to examine associations among critical thinking, sustainable behaviors, and emotional avoidance, both at the total scale level and across subscales.

To test the hypotheses, multiple regression analyses were conducted to examine the contributions of critical thinking and emotional avoidance to sustainable behaviors.

We conducted two simple linear regressions (H1, H2) and one multiple linear regression including both predictors entered simultaneously. Additional models included age, gender, and education as covariates. The final model assessed the independent contribution of each predictor to sustainable behaviors. In additional analyses, age, gender, and educational level were included as covariates to examine the robustness of the regression results.

Effect sizes were reported using r, R^2^, and standardized regression coefficients (β). Statistical significance was set at α = 0.05 (two-tailed), and 95% confidence intervals were reported for regression coefficients.

This analytical strategy enhances the interpretability and replicability of the findings by clearly linking statistical procedures to the study hypotheses and theoretical framework.

## Results

Sample characteristics

Participants ranged in age from 18 to 64 years (*M* = 31.50, *SD* = 11.69). On average, households consisted of 3.30 members (*SD* = 1.27; range = 1-7). Most participants identified as female (*n* = 184, 59.5%), followed by male (*n* = 119, 38.5%), and a small percentage identified as non-binary (*n* = 6, 1.9%). Regarding occupation, the largest group was workers (33.6%), followed by students (31.4%). Additional demographic information is presented in Table 1.

**Table 1:** Demographic characteristics of the sample.

Variable	*n*	%
*Gender*		
Female	184	59.5
Male	119	38.5
Not binary	6	1.9
*Age* (M, SD)		31.50 (11.69)
*Occupation*		
Student	98	31.4
Unemployed	30	9.6
Homemaker	12	3.9
Worker	105	33.6
Employee	36	11.5
Healthcare professional	20	6.4
Other	11	3.6
*Economic independence*		
No	144	46.6
Yes	165	53.4
*Living alone*		
No	249	80.6
Yes	60	19.4

Descriptive Statistics

Table 2 reports the descriptive statistics for the main study variables. For each scale, means, standard deviations, and observed ranges are presented.

**Table 2:** Descriptive Statistics of Main Variables (N = 309).

Scale	M (SD)	Observed range
*Critical Thinking Attitude Scale (CTAS)*		
Systematicity	48.84 (7.84)	18–63
Truth-seeking/open-mindedness	33.46 (5.68)	15–42
Analyticity	21.21 (3.68)	11–28
Intellectual curiosity	37.38 (6.54)	16–49
Critical thinking (total)	140.89 (20.83)	72–182
*Sustainable Behaviors Scale (SBS)*		
Sustainable purchasing and awareness	38.51 (7.70)	13–56
Reuse and recycling	22.01 (5.04)	4–28
Reduction (e.g., energy, water)	21.94 (3.93)	6–28
Walking and public transportation	9.32 (3.43)	2–14
Sustainable behaviors (total)	91.79 (16.38)	25–126
*Brief Experiential Avoidance Questionnaire (BEAQ)*		
Tendency to avoid emotions	37.53 (7.93)	10–60
Lack of emotional awareness during activity	16.25 (4.53)	5–30
Emotional avoidance (total)	53.78 (11.29)	15–90

*N* = *309. For each scale and subscale, means, standard deviations, and observed ranges are reported. Theoretical ranges were computed based on the response formats and number of items for each measure: CTAS (26–182), SBS (18–126), BEAQ (15–90). All distributions had skewness and kurtosis values between* −*1 and* +*1, indicating approximate normality. Internal consistency was adequate across measures and subscales (Cronbach's* α *ranged from 0.78 to 0.89)*.

### Correlations

Pearson's correlations were first conducted with the global measures and subsequently with the subscales.

Table 3 shows that critical thinking was positively and significantly associated with sustainable behaviors (*r* = 0.34, *p* < 0.001), supporting the hypothesis of a direct relationship. In contrast, emotional avoidance was negatively correlated with critical thinking (*r* = −0.37, *p* < 0.05), but not significantly associated with sustainable behaviors (*r* = −0.08, *p* = 0.143).

**Table 3:** Correlations among critical thinking, sustainable behaviors, and emotional avoidance (N = 309).

Variable	1	2	3
1. Critical thinking (total)	-		
2. Sustainable behaviors (total)	0.34^**^	-	
3. Emotional avoidance (total)	−0.37^*^	−0.08	-

*Values represent Pearson's r. p*<0.05^*^, p<0.01^**^.

Further analyses are presented in Table 4. Sustainable behavior total scores were strongly correlated with all its subscales: purchasing and awareness (*r* = 0.91, *p* < 0.01), reuse and recycling (*r* = 0.84, *p* < 0.01), reduction (*r* = 0.78, *p* < 0.01), and transportation (*r* = 0.61, *p* < 0.01), reflecting strong internal coherence.

**Table 4:** Correlations among all subscales of critical thinking, sustainable behaviors, and emotional avoidance (N = 309).

	Variable	1	2	3	4	5	6	7	8	9	10	11	12	13
1	Sustainable behaviors (total)	-												
2	Purchasing/awareness	0.91^**^	-											
3	Reuse/recycling	0.84^**^	0.68^**^	-										
4	Reduction (energy, water)	0.78^**^	0.59^**^	0.59^**^	-									
5	Walking & public transportation	0.61^**^	0.42^**^	0.37^**^	0.39^**^	-								
6	Critical thinking (total)	0.34^**^	0.30^**^	0.31^**^	0.36^**^	0.08	-							
7	Systematicity	0.30^**^	0.29^**^	0.31^**^	0.31^**^	0.07	0.90^**^	-						
8	Truth-seeking/Open-mindedness	0.31^**^	0.21^**^	0.25^**^	0.35^**^	0.08	0.90^**^	0.72^**^	-					
9	Analyticity	0.36^**^	0.32^**^	0.35^**^	0.30^**^	0.10	0.83^**^	0.72^**^	0.69^**^	-				
10	Intellectual curiosity	0.08	0.08	0.08	0.03	0.10	0.87^**^	0.63^**^	0.75^**^	0.62^**^	-			
11	Emotional avoidance (total)	−0.11	0.02	−0.11	−0.14^*^	−0.13^*^	−0.37^*^	−0.20^**^	−0.11	−0.03	−0.08	-		
12	Tendency to avoid emotions	−0.04	0.05	−0.04	−0.09	−0.15^**^	−0.06	−0.12^*^	−0.04	0.05	−0.05	0.95^**^	-	
13	Lack of emotional awareness	−0.27^**^	−0.23^**^	−0.22^**^	−0.18^**^	−0.05	−0.23^**^	−0.31^**^	−0.19^**^	−0.15^*^	−0.13^*^	0.83^**^	0.61^**^	-

Values are Pearson's correlation coecients (r). Cells above the diagonal are left blank; * *p* < 0.05, ** *p* < 0.01.

Sustainable behaviors (total) were also positively correlated with critical thinking (total; *r* = 0.34, *p* < 0.01). At the subscale level, systematicity (*r* = 0.30, *p* < 0.01), truth-seeking/open-mindedness (r = 0.31, *p* < 0.01), and analyticity (*r* = 0.36, *p* < 0.01) were positively associated, whereas intellectual curiosity was not significant (*r* = 0.08, ns). This suggests that analytic and evaluative aspects of critical thinking are most relevant for sustainable actions.

Emotional avoidance total scores were not significantly related to sustainable behaviors (r = −0.08, ns). However, lack of emotional awareness during activity showed consistent negative correlations with sustainable behaviors (total: *r* = −0.27, *p* < 0.01) and with all its subscales, indicating that reduced emotional awareness is linked to lower ecological engagement.

### Hypothesis testing

To test the hypotheses, a series of simple linear regression analyses and a multiple linear regression analysis were conducted.

To test H1, a simple linear regression analysis was conducted with total critical thinking as the predictor and total sustainable behaviors as the outcome. The model was significant, explaining 12% of the variance in sustainable behaviors, *R*^2^ = 0.12, F _(1, 307)_ = 41.70, *p* < 0.001. Critical thinking emerged as a positive predictor, β = 0.34, *t* = 6.46, *p* < 0.001, 95% CI [0.23, 0.45], supporting H1.

To test H2, a second simple linear regression analysis was performed using lack of emotional awareness during activity as the predictor and sustainable behaviors as the outcome. This model was also significant, explaining 7% of the variance, *R*^2^ =0.07, F _(1, 307)_ = 22.90, *p* < 0.001. Lack of emotional awareness negatively predicted sustainable behaviors, β = −0.27, *t* = −4.78, *p* < 0.001, 95% CI [−0.38, −0.15], supporting H2.

Finally, a multiple regression model including both critical thinking and lack of emotional awareness was conducted to examine their independent contributions. The overall model was significant, *R*^2^ = 0.16, *F*_(2, 306)_ = 28.90, *p* < 0.001. Both predictors remained significant: critical thinking (β = 0.27, *p* < 0.001) and lack of emotional awareness (β = −0.22, *p* < 0.001).

**Figure 1:**
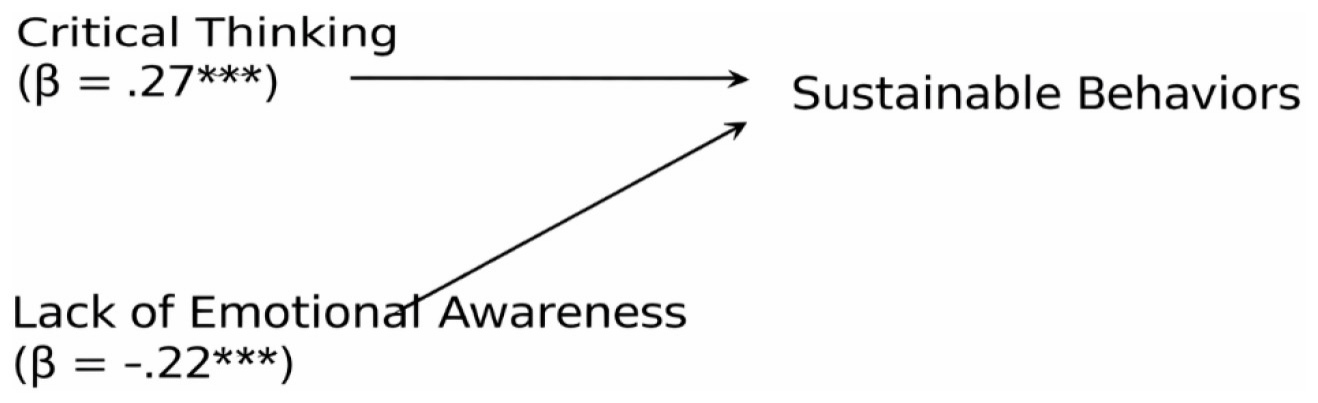
Schematic representation of the regression model predicting sustainable behaviors. Critical thinking shows a positive predictive effect, whereas lack of emotional awareness during activity shows a negative predictive effect. Standardized regression coefficients (β) are reported. **p <0.001.

As illustrated in Figure 1, critical thinking showed a positive predictive path toward sustainable behaviors, whereas lack of emotional awareness showed a negative independent effect.

When age, gender, and educational level were included as covariates in additional analyses, the results remained substantively unchanged, and both critical thinking and lack of emotional awareness continued to significantly predict sustainable behaviors.

These findings suggest that critical thinking fosters sustainable behaviors even after controlling for emotional avoidance, while reduced emotional awareness independently hinders such behaviors.

## Discussion

The present study examined the role of critical thinking and emotional processes in predicting sustainable behaviors within a Theory of Planned Behavior–informed (TPB; Ajzen, 1991, 2005, 2012) perspective. Results confirmed both hypotheses: critical thinking positively predicted sustainable behaviors (H1), whereas lack of emotional awareness negatively predicted them (H2). Importantly, the combined model showed that these dimensions independently and complementarily contributed to sustainable behaviors, consistent with their role as cognitive–emotional resources associated with engagement in pro-environmental behavior.

### Critical thinking and sustainable behaviors

Consistent with H1, individuals with higher critical thinking reported stronger engagement in sustainable behaviors, aligning with prior research highlighting the role of systematic analysis and evaluation in informed decision-making (Willingham, 2007; Sternberg, 2018; Paul and Elder, 2020; Fabio et al., 2025b). In particular, the components of analyticity and truth-seeking/open-mindedness were the most relevant, indicating that the ability to systematically evaluate information and integrate multiple perspectives is crucial for translating environmental knowledge into action.

These findings are consistent with prior research on critical thinking and informed decision-making (Willingham, 2007; Sternberg, 2018; Paul and Elder, 2020; Facione, 2015; Fabio et al., 2025a) and support the view that critical thinking is associated with how individuals evaluate and integrate environmental information, thereby facilitating engagement in sustainable behaviors within a TPB-informed framework.

### Emotional avoidance and sustainable behaviors

Although total emotional avoidance was not related to sustainable behaviors, the subscale lack of emotional awareness during activity showed consistent negative associations with all ecological outcomes. This suggests that the ability to recognize and process emotions in the moment of action is critical for motivating ecological engagement. Reduced emotional awareness may limit the affective elaboration of environmental beliefs, decreasing their translation into attitudes and reducing motivation for action, consistent with prior research on climate-related emotions (Lertzman, 2015; Clayton, 2020; Norgaard, 2011; Stoknes, 2015; Reser and Bradley, 2020; Ojala, 2012; Stanley et al., 2021).

### Additive and interactive effects

The results indicate predominantly additive effects: critical thinking and emotional awareness independently contribute to sustainable behaviors. Together, these findings highlight the complementary role of cognitive and emotional resources in supporting engagement in sustainable practices, without implying synergistic or interactive effects beyond those directly tested.

### Integration with theory

By conceptualizing critical thinking and emotional awareness as upstream cognitive–emotional resources, the present study adopts a TPB-informed perspective to highlight psychological processes that may precede and support sustainable behavior. Individuals with greater cognitive–emotional resources may evaluate and integrate environmental information more effectively, resulting in more consistent engagement in sustainable behaviors. Rather than extending or empirically testing the TPB model, the present findings are consistent with TPB-based perspectives that emphasize the role of cognitive and affective processes in environmentally relevant behavior (Halpern and Dunn, 2022; Fabio et al., 2025a). Importantly, the robustness of the findings was supported by additional analyses controlling for key demographic variables, suggesting that the observed associations are not attributable to age, gender, or educational differences.

## Limitations

Despite the promising results, several limitations must be acknowledged to contextualize the findings within the theoretical framework of the study. A key limitation of the present study is the absence of direct measures of core Theory of Planned Behavior constructs, such as attitudes, subjective norms, perceived behavioral control, and behavioral intentions. Consequently, conclusions are limited to self-reported sustainable behaviors and do not allow for empirical testing or extension of the TPB. Future research should incorporate these constructs to evaluate whether critical thinking and emotional awareness contribute to TPB pathways and offer incremental explanatory power within a full TPB framework.

Cross-sectional design. The correlational nature of this study prevents causal inferences, limiting our ability to confirm whether critical thinking and emotional awareness actively drive sustainable behaviors, by the present TPB-informed perspective. Although our hypotheses (H1 and H2) are supported, longitudinal or experimental designs are needed to test the temporal and causal pathways through which cognitive–emotional resources shape pro-environmental attitudes and behaviors.

Common-method bias. All variables were assessed via self-report questionnaires, which introduces the risk of common-method variance. Individuals with higher self-perceived critical thinking or emotional awareness might overreport sustainable behaviors due to social desirability or self-enhancement tendencies, inflating correlations and potentially exaggerating the independent contributions of cognition and emotion. Such bias could obscure the true strength and nature of the relationships hypothesized in the TPB framework. Future research could mitigate these effects by incorporating behavioral measures, ecological momentary assessment, or multi-informant reports, thereby providing more robust tests of the proposed mechanisms.

Construct overlap and multicollinearity. Certain subscales, such as Analyticity and Systematicity within critical thinking, may share conceptual variance, while components of emotional avoidance may also correlate. This overlap can reduce the precision of estimating unique effects and complicate the interpretation of which cognitive or emotional facets truly drive sustainable behaviors. For instance, the observed additive and interactive effects might partly reflect shared variance rather than distinct theoretical constructs. Advanced statistical approaches, such as structural equation modeling or bifactor models, could clarify the distinct roles of these cognitive–emotional resources within the TPB model.

Sample characteristics and generalizability. The sample was predominantly young, educated, and Italian, which may limit the applicability of the findings to other age groups, cultures, or educational backgrounds. Replication in more diverse populations is necessary to verify whether the observed cognitive–emotional pathways generalize across different contexts.

Unmeasured variables. Other theoretically relevant factors, such as social norms, personal values, environmental knowledge, and situational constraints, were not assessed. These variables could moderate or mediate the relationships between cognitive–emotional resources and sustainable behaviors, affecting the explanatory power of the TPB extension. Future studies should consider integrating these factors to test full TPB models.

By addressing these limitations, future research can provide a more precise and theoretically grounded understanding of how critical thinking and emotional awareness function as complementary drivers of sustainable behaviors, reinforcing the TPB framework and informing effective interventions.

### Practical implications and future directions

The present findings offer both practical and theoretical insights for promoting sustainable behaviors through the integration of cognitive and emotional resources.

Practical Implications. Interventions to enhance ecological engagement should combine critical thinking and emotional awareness training, tailored to specific contexts. For example:

Educational settings (schools and universities): Programs could include structured exercises in analytic reasoning and truth-seeking, alongside workshops on emotional recognition and regulation during environmental decision-making.

Organizational or community initiatives: Employee or citizen engagement programs can incorporate scenario-based problem solving with feedback on cognitive evaluation, coupled with guided reflection on emotional responses to environmental challenges.

These targeted applications ensure that critical thinking skills are applied in realistic decision-making contexts, while emotional awareness supports motivation and affective processing, reflecting the additive and complementary influence of cognitive–emotional resources observed in this study. Interventions that focus on only one dimension may be less effective, underscoring the value of integrated approaches.

Future Research Directions. Beyond methodological improvements (e.g., behavioral observations, longitudinal designs), future studies could explicitly test TPB-based mediational and moderational pathways. For instance:

Mediators: Examine whether the effects of critical thinking and emotional awareness on sustainable behaviors are mediated by pro-environmental attitudes, perceived behavioral control, or intention, consistent with the TPB framework.

Moderators: Test whether social norms, personal values, or situational constraints strengthen or weaken the influence of cognitive–emotional resources on intentions and behaviors.

Advanced modeling: Structural equation modeling or path analysis can be employed to simultaneously estimate these pathways, clarifying the mechanisms through which cognitive–emotional resources shape sustainable behaviors.

Theoretical advancement can also arise from investigating the interaction of cognition and emotion: future studies could explore whether high emotional awareness enhances the translation of analytic reasoning into behavioral intentions, or whether specific combinations of cognitive–emotional traits predict different types of pro-environmental actions. Such approaches move beyond descriptive associations, directly linking the findings to TPB mechanisms and offering testable hypotheses for future research.

Overall, these insights support the development of context-specific, theory-driven interventions and provide a framework for future studies to advance understanding of the cognitive–emotional underpinnings of sustainable behavior (Balaskas, 2024).

## Data Availability

The original contributions presented in the study are included in the article/supplementary material, further inquiries can be directed to the corresponding author.

## References

[B1] Ahuja J. Yadav M. Sergio R. P. (2023). Green leadership and pro-environmental behaviour: a moderated mediation model with rewards, self-efficacy and training. Int. J. Ethics Syst. 39, 481–501. doi: 10.1108/IJOES-02-2022-0041

[B2] Ajzen I. (1991). The theory of planned behavior. Organ. Behav. Hum. Decis. Process. 50, 179–211. doi: 10.1016/0749-5978(91)90020-T

[B3] Ajzen I. (2005). Attitudes, Personality, and Behavior, 2nd Edn. Maidenhead: Open University Press/McGraw-Hill.

[B4] Ajzen I. (2012). “The theory of planned behavior,” in Handbook of Theories of Social Psychology Vol 1, eds. P. A. M. Van Lange, A. W. Kruglanski, and E. T. Higgins (Thousand Oaks, CA: SAGE Publications), 438–459. doi: 10.4135/9781446249215.n22

[B5] Balaskas S. (2024). HEXACO traits, emotions, and social media in shaping climate action and sustainable consumption: the mediating role of climate change worry. Psychol. Int. 6, 937–976. doi: 10.3390/psycholint6040060

[B6] Bosnjak M. Ajzen I. Schmidt P. (2020). The theory of planned behavior: selected recent advances and applications. Eur. J. Psychol. 16, 352–356. doi: 10.5964/ejop.v16i3.3107 33680187 PMC7909498

[B7] Clayton S. (2020). Climate anxiety: psychological responses to climate change. J. Anxiety Disord. 74:102263. doi: 10.1016/j.janxdis.2020.102263 32623280

[B8] Fabio R. A. Croce A. (2024). The role of peace attitudes on sustainable behaviors: an exploratory study. Behav. Sci. 14:120. doi: 10.3390/bs14020120 38392473 PMC10886167

[B9] Fabio R. A. Croce A. Calabrese C. (2024). Construction and psychometric properties of the sustainable behavior questionnaire among Italian adults. *Sustain. Dev*. 32, 4374–4384. doi: 10.1002/sd.2902

[B10] Fabio R. A. Croce A. Calabrese C. (2025b). Bridging the green attitude–behavior gap. J. Sustain. Res. 7:e250059. doi: 10.20900/jsr20250059

[B11] Fabio R. A. Plebe A. Ascone C. Suriano R. (2025a). Psychometric properties and validation of critical reasoning assessment. Pers. Individ. Dif. 246:113344. doi: 10.1016/j.paid.2025.113344

[B12] Facione P. (2015). Critical Thinking: What It Is and Why It Counts. San Jose, CA: Insight Assessment.

[B13] Facione P. A. Sanchez C. A. Facione N. C. Gainen J. (1995). The disposition toward critical thinking. J. Gen. Educ. 44, 1–25.

[B14] Faul F. Erdfelder E. Buchner A. Lang A.-G. (2009). Statistical power analyses using G*Power 3.1: Tests for correlation and regression analyses. Behav. Res. Methods 41, 1149–1160. doi: 10.3758/BRM.41.4.1149 19897823

[B15] Felix S. M. Clayton S. (2025). Describing teachers' environmental identity as part of education for sustainable development. Environ. Educ. Res. 2, 1–18. doi: 10.1080/13504622.2025.2482740

[B16] Gámez W. Chmielewski M. Kotov R. Ruggero C. Suzuki N. Watson D. (2014). The brief experiential avoidance questionnaire: development and initial validation. Psychol. Assess. 26, 35–45. doi: 10.1037/a0034473 24059474

[B17] Halpern D. F. Dunn D. S. (2022). Thought and Knowledge: An Introduction to Critical Thinking, 6th Edn. Milton Park: Routledge. doi: 10.4324/9781003025412

[B18] Howard G. S. (2000). Adapting human lifestyles for the 21st century. Am. Psychol. 55, 509–515. doi: 10.1037/0003-066X.55.5.509 10842431

[B19] Javaid M. Kumari K. Khan S. N. Jaaron A. A. M. Shaikh Z. (2023). Leader green behavior as an outcome of followers' critical thinking and active engagement: the moderating role of pro-environmental behavior. Leadersh. Organ. Dev. J. 44, 218–239. doi: 10.1108/LODJ-07-2021-0361

[B20] Lamb S. Maire Q. Doecke E. (2017). Key Skills for the 21st Century: An Evidence-Based Review. Centre for International Research on Education Systems (CIRES), Victoria University.

[B21] Lange F. Dewitte S. (2019). Measuring pro-environmental behavior: review and recommendations. J. Environ. Psychol. 63, 92–100. doi: 10.1016/j.jenvp.2019.04.009

[B22] Lertzman R. (2015). Environmental Melancholia: Psychoanalytic Dimensions of Engagement. Milton Park: Routledge. doi: 10.4324/9781315851853

[B23] Luchs M. G. Naylor R. W. Raghunathan R. (2010). The sustainability liability: potential negative effects of ethicality on product preference. J. Mark. 74, 18–32. doi: 10.1509/jmkg.74.5.018

[B24] Nawawi S. Johari A. Siburian J. Anggereini E. (2024). The transformative power of challenge-based learning in cultivating 21st-century skills. Int. J. Educ. Res. Rev. 7:84243. doi: 10.23887/ijerr.v7i3.84243

[B25] Nielsen K. S. Clayton S. Stern P. C. Dietz T. (2020). How psychology can help limit climate change. Am. Psychol. 76, 130–144. doi: 10.1037/amp0000624 32202817

[B26] Norgaard K. M. (2011). Living in denial: Climate change, emotions, and everyday life. Cambridge, MA: MIT Press. doi: 10.7551/mitpress/9780262015448.001.0001 23673764

[B27] Ojala M. (2012). Hope and climate change: the importance of hope for environmental engagement among young people. Environ. Educ. Res. 18, 625–642. doi: 10.1080/13504622.2011.637157

[B28] Otto S. Kaiser F. G. Arnold O. (2014). The critical challenge of climate change for psychology: preventing rebound and promoting more individual responsibility. Eur. Psychol. 19, 96–106. doi: 10.1027/1016-9040/a000182

[B29] Paul R. Elder L. (2020). The Miniature Guide to Critical Thinking Concepts and Tools, 8th Edn. Santa Barbara, CA: The Foundation for Critical Thinking. doi: 10.5771/9781538133842

[B30] Profetto-McGrath J. Smith K. B. Hugo K. Patel A. Dussault B. (2009). Nurse educators' critical thinking dispositions and research utilization. Nurse Educ. Pract. 9, 199–208. doi: 10.1016/j.nepr.2008.06.003 18701349

[B31] Reser J. P. Bradley G. L. (2020). The nature, significance, and influence of perceived personal experience of climate change. Wiley Interdiscip. Rev. Climate Change 11:e668. doi: 10.1002/wcc.668

[B32] Rhodes T. L. (Ed.). (2010). Assessing Outcomes and Improving Achievement: Tips and Tools for Using Rubrics. Washington, DC: Association of American Colleges and Universities.

[B33] Ripple W. J. Wolf C. Newsome T. M. Galetti M. Alamgir M. Crist E. . (2017). World scientists' warning to humanity: a second notice. BioScience 67, 1026–1028. doi: 10.1093/biosci/bix125

[B34] Stanley S. Hogg T. L. Leviston Z. Walker I. (2021). From anger to action: Differential impacts of eco-anxiety, eco-depression, and eco-anger on climate action and wellbeing. J. Clim. Chang. Health 1:100003. doi: 10.1016/j.joclim.2021.100003

[B35] Sternberg R. J. (2018). “The triarchic theory of successful intelligence,” in Contemporary Intellectual Assessment: Theories, Tests, and Issues, 4th Edn.eds. D. P. Flanagan and E. M. McDonough (New York, NY: The Guilford Press), 174–194.

[B36] Stoknes P. E. (2015). What We Think About When We Try Not to Think About Global Warming. White River Junction, VT: Chelsea Green Publishing.

[B37] Tilikidou I. Delistavrou A. (2014). Pro-environmental purchasing behaviour during the economic crisis. Market. Intel. Plann. 32, 160–173. doi: 10.1108/MIP-10-2012-0103

[B38] Verburgh A. François S. Elen J. Janssen R. (2013). The assessment of critical thinking critically assessed in higher education: a validation study of the CCTT and the HCTA. Educ. Res. Int. 2013:198920. doi: 10.1155/2013/198920

[B39] Vesely S. Klöckner C. A. Carrus G. Tiberio L. Caffaro F. Biresselioglu M. E. . (2022). Norms, prices, and commitment: a comprehensive overview of field experiments in the energy domain and treatment effect moderators. Front. Psychol. 13:967318. doi: 10.3389/fpsyg.2022.967318 36425813 PMC9680531

[B40] Wang B. Udall A. M. (2023). Sustainable consumer behaviors: The effects of identity, environment value and marketing promotion. Sustainability 15:1129. doi: 10.3390/su15021129

[B41] White M. P. Alcock I. Grellier J. Wheeler B. W. Hartig T. Warber S. L. . (2019). Spending at least 120 minutes a week in nature is associated with good health and wellbeing. Sci. Rep. 9:7730. doi: 10.1038/s41598-019-44097-3 31197192 PMC6565732

[B42] Willingham D. T. (2007). Critical thinking: why is it so hard to teach? Arts Educ. Policy Rev. 109, 21–32. doi: 10.3200/AEPR.109.4.21-32

